# Aggressive behaviors and associated factors in Chinese left-behind adolescents: a cross-sectional study

**DOI:** 10.1186/s12887-022-03736-x

**Published:** 2022-11-23

**Authors:** Fang Yang, Zhiyu Jin, Jing He, Bingsong Han, Xinyuan Huang, Kun Chen, Jiana Wang

**Affiliations:** 1 Teaching and Research Section of School SportSchool of Physical Education, Shenyang Sport University, Shenyang, China; 2 Department of Social Medicine, School of Health Management, China Medical University, No. 77 Puhe Road, Shenyang North New Area, Shenyang, Liaoning 110122 People’s Republic of China

**Keywords:** Aggressive behavior, Left-behind adolescent, Cross-sectional study, China

## Abstract

**Background:**

To examine whether the levels of aggressive behaviors and other individual and contextual variables differ between left-behind adolescents (LBA) and not left-behind adolescents (NLBA) and explore associations between aggression and other constructs among them.

**Methods:**

A cross-sectional study was conducted and 4530 school adolescents aged 9–18 years in north and south of China were randomly selected. The levels of aggressive behavior, personality and family and classroom environment were compared between LBA and NLBA and also the associated factors of aggression.

**Results:**

The total scores of aggressive behaviors were 6.33 ± 6.35 (Mean ± SD) in LBA and 5.78 ± 6.16 (Mean ± SD) in NLBA. Multiple linear regression models revealed that neuroticism and psychoticism were positively associated with aggressive behaviors for LBA with similar results of NLBA. Cohesion was negatively associated with aggressive behaviors, and conflict and achievement had positive effects in NLBA. Organization had a negative effect in LBA. Uncertainty and dissatisfaction had positive effects on aggression both in LBA and NLBA.

**Conclusion:**

This study found a slightly higher level of aggressive behaviors in LBA comparing with NLBA. Personality was the mainly associated factor of aggression, but class-based interventions were more practical for aggressive behaviors in Chinese LBA.

## Introduction

Over the years, violence is one of the most concerned global public health problems, and aggression is a common act of violence in children and adolescents. Adolescence is an increasing period of severe aggressive and violent behaviors. The report of Chinese Ministry of Public Security shows that the number of juvenile crimes in China has risen by an average of 13% per year. The total number of juvenile crimes has accounted for more than 70% of the total criminal crimes in China, and most criminal youth have shown aggressive behaviors before age of 13 [1]. Early persistent aggressive behaviors, if not intervene timely, might lead to conduct problems, school failure, antisocial personality, and even violent crimes, and bring about serious public health burden [2]. Moreover, in Chinese culture, youth’s aggressive behaviors might be more likely to be unacceptable by others such as parents, peers and teachers compared to western culture due to the influence of Collectivism and Confucianism which value group harmony [3]. Therefore, it is urgent to pay more attention to Chinese adolescents’ aggressive behaviors.

Rapid urbanization in China encouraged large numbers of rural residents to flock to cities for better job opportunities, leaving their children at home and creating a potentially vulnerable subpopulation named left-behind children. The definition of left-behind children is those who, under 18 years old, stay in their hometowns while one or both parents work away from home for at least six months [4]. LBA refers to the adolescents in left-behind children. NLBA refers to the not left-behind adolescents comparing with LBA. Foreign LBA with small numbers mainly exists in developing countries, forcing their citizens seeking work abroad due to the unemployment and poverty [5–7]. China has the largest number of LBA due to the unprecedented economic growth and the following nationwide population mobility. There were approximately 61 million left-behind children in China, account for 37.7% of the rural children and 22% of all children in the whole country [8]. Between 1993 and 2005, there were only hundreds of literatures about LBA. By the end of 2012, the numbers of literatures already reached 6611 which mainly focus on nutrition, depression and anxiety, school achievement and parental rearing [9]. Two latest meta-analysis studies found that LBA were suffering from more mental health problems [10, 11]. Until recently, attention was focused on the behavior of LBA. Totally, LBA have two extreme behavioral problems, being injured, or have violent behaviors. Earlier separation from their parents would increase the risk of further generating aggressive and violent behaviors. Long term lack of parental care makes LBA yearn for the attention from other people. On the other hand, they can also relieve their negative feelings through some special acts such as aggressive behaviors. One study showed that 31.24% LBA had aggressive behaviors and left behind may be a risk factor of aggression [12]. Therefore, it is extremely essential to understand aggressive behaviors in this vulnerable population aiming at reducing delinquent conduct and maintaining social stability.

The General Aggression Model (GAM) is generally recognized a theoretical model to explain what the aggression constructs and how it conducts. It considers that input variables (individual factors and environmental factors) work together on individuals and ultimately output aggressive behaviors. Based on it, the psychology of aggression generally explores the influences on aggression from two aspects of individual factors and environment. Personality can help understand why some people are more likely to develop improper behaviors [13]. Those who showed poor disposition to experience fear, lack of empathy for others, might have less control over behaviors which can further lead to high proactive aggression [14]. It was argued that personality factors were the key predictors for aggressive behaviors [15]. The general aggression model (GAM) considers that some traits like neuroticism and agreeableness can aggravate or protect the generation of aggression through affecting social cognitive and affective processes [16]. There were many evidences showed the relationship between aggression and traits in either normal adult [17], normal youth [18] or offender population [19], indicating that some personality traits reinforce aggressive beliefs and scripts. However, few studies explored how the personality works on LBA’s aggression. Moreover, aggression increases more over time in risky contextual environment than in supportive one [20]. Many studies demonstrated family functions such as parenting style and parent–child cohesion had significant influences on adolescents’ mental development [10, 21, 22]. In China, class is the essential unit of student activity, with fixed classrooms and teachers and continually affects students’ behavior to a greater extent than that in western school. Especially for LBA, positive classroom environment and teacher-student relationships might make up the lack of parental accompany. One review summarized that LBA had poorer performance and worse interaction with teachers at school than common adolescents [23]. Different types of environments have different impacts on the aggressiveness between LBA and NLBA. Compared with NLBA, LBA is more affected by the classroom environment [24]. The negative teacher-student relationships might lead to problem behaviors whereas the positive classroom environment and interpersonal relationships would be protective factors of aggressive behaviors [25]. Though the link between personality and aggression or environment and aggression have received considerable attention, only few studies found both personality and environment effects on adolescents’ behavioral problems [26]. Therefore, it is necessary to explore both personality and environmental effects and compare different effects between LBA and NLBA.

The current study examined whether aggressive behaviors and other individual and contextual variables differ in a large sample involving comparisons between LBA and NLBA and explored associations between aggression and other constructs. We addressed the following hypotheses: (1) There is a difference in the level of aggressive behavior between LBA and NLBA; (2) while individual personality and family environment differences accounted for much of the total variation in aggression of LBA, the classroom environment indicated by average teacher-student interactions would further contribute to the variation; and (3) classroom environment and family environment contribute differently to the aggressive behavior of LBA and NLBA.

## Materials and methods

### Study design and study sample

This study was conducted in two Provinces, Liaoning, and Anhui during the period of November–December 2010. Liaoning is located in north China, and Anhui in eastern. There are 48 million population in Liaoning province, and 60 million in Anhui. The average income in these two provinces was almost in the same level as the average national level in China. Consult relevant research and investigation, the detection rate of behavioral problems in children and adolescents was 30%, and the allowable error and confidence interval were 2% and 95%, respectively [27]. The effective response rate was calculated based on 70%. The sample size required in each province was at least 2858, and that in two provinces was at least 5716.

$$\text{n} = \frac{{U}_{\alpha }\pi \left(1-\pi \right)}{{\delta }^{2}}$$

Twenty-seven schools including 11 primary schools (grades 4–6), 9 junior high school (grades 7–9) and 7 senior schools (grades 10–12) were randomly selected. One-third of the classes were randomly selected from each grade, and, according to the estimated sample size, 25 students in each selected class were randomly selected, with an equal number of boys and girls. Finally, a total of 6075 students were chosen for inclusion in this study. All the participants and their parents were well informed about the contents and aims of this study. After obtaining written consent to conduct the survey, a set of questionnaires, including the Child Behavior Checklist (CBCL), Eysenck Personality Questionnaire (EPQ), Family Environment Scale (FES), and Questionnaire on Teacher Interaction (QTI), and a request for personal information were distributed to the 6075 participants. Of the 5427 students who returned questionnaires, 897 were excluded from the study due to incorrect information or missing data. Comparing the excluded respondents and those who were included, there were no significant differences in gender and age (*P* > 0.05). Therefore, we have reasons to believe that the excluded students can match the included students in basic characteristics. The final study subjects consisted of 4530 Chinese adolescents attending school, and the effective response rate was 74.6%. We categorized LBA and NLBA by the question: ‘Do your parents or one of them ever or now migrate to another place over six months?’ according to the definition of LBA who had been left behind by at least one parent for > 6 months. As a result, 1078 students were treated as LBA.

### Measurements

The current study used the subscale “Aggressive Behavior” of the Chinese versions of the Child Behavior Checklist (CBCL) to measure the level of aggressive behavior in our participants. The aggressive behavior of children aged 4–18 was assessed, including 23 items in the 6–11-year-old aggressive behavior scale and 22 items in the 12–18-year-old aggressive behavior scale according to the calculation principle of the CBCL [28]. Each item has three response categories: (0) not true (as far as you know), (1) somewhat or sometimes true, (2) very true or often true. Higher scores denote greater problems. A good reliability and validity of the CBCL was confirmed for the Chinese version [29]. The Cronbach’s alpha was above 0.80 for the aggression subscale in this study.

Personality was assessed using an adapted version of the EPQ for children [30]. This version has 88 true–false items and includes four subscales: extraversion (E), psychoticism (P), neuroticism (N), and lie (L). The Cronbach’s alpha was 0.704. EPQ has a good reliability and validity in Chinese population [31, 32].

The FES has 90 true–false items and includes ten subscales: cohesion, expressiveness, conflict, independence, achievement, intellectual-cultural orientation, active-recreational orientation, moral-religious emphasis, organization, and control [33]. Seven subscales were used in this study due to the inapplicability of the three excluded subscales (expressiveness, independence, and moral-religious subscales) when used in the Chinese population [34]. The Cronbach’s alpha was 0.83. It has been found a good reliability and validity in China [31, 32].

Teacher-student interaction was measured by the QTI, which was developed to map interpersonal teacher behavior with eight subscales: leadership, helpful/friendly, understanding, student responsibility/freedom, uncertainty, dissatisfaction, admonishing, and strict. This study used students’ responses to the shorter 48-item Chinese revised version of the QTI (six items for each scale), which was appropriate for primary students [35]. It has been widely used among Chinese populations with good reliability and validity [36] and the Cronbach’s alpha was 0.7262.

Confounding factors included students’ gender, age, only child and socioeconomic status (SES). SES was determined by the parents’ highest education level and was divided into low, middle, and high SES.

### Statistical analysis

Descriptive statistics were carried out for socio-demographic data. Independent t-and chi-squared tests were used to compare the socio-demographic factors and aggressive behaviors between LBA and NLBA. Pearson’s correlation was used for the relations between EPQ, FES, QTI and aggressive behavior scores. The residuals of linear regression model obey normal distribution. Multiple linear regression analysis was used to estimate the associated factors of aggressive behaviors. All variables related to aggressive behaviors in univariate analysis (*p* < 0.05) were entered into the model. All the statistical analyses were performed with SPSS for Windows (version 20.0), with two-tailed probability value of < 0.05 considered to be statistically significant.

## Results

The participants consisted of 1078 LBA and 3452 NLBA. The average ages were 13.58 ± 2.70 in LBA and 13.28 ± 2.63 in NLBA respectively (*p* < 0.05). The distribution of other socio-demographic characteristics between the LBA and NLBA are shown in Table 1.

**Table 1:** Comparison of demographics between LBA and NLBA

**Variable**	**LBA (*n* = 1078)** % (n)	**NLBA (*n* = 3452)** % (n)	***P*-value**
Gender			0.005
Boy	49.5(534)	44.6(1540)	
Girl	50.5(544)	55.4(1912)	
SES			0.000
High	14.8(160)	19.7(681)	
Middle	35.8(386)	37.1(1281)	
Low	49.4(532)	43.2(1490)	
Only child			0.000
Yes	46.1(497)	54.8(1891)	
No	53.9(581)	45.2(1561)	

*Abbreviation*: *LBA* Left-behind adolescent, *NLBA* Not-left-behind adolescent, *SES* Socioeconomic status

Table 2 compared the scores of aggressive behaviors, EPQ, FES and QTI between LBA and NLBA. LBA had a slightly higher aggression score than NLBA (*p* = 0.011). Meanwhile, LBA had a significantly lower extraversion score but higher psychoticism and neuroticism scores. LBA also had relatively lower family cohesion, achievement levels but higher conflict and control levels in FES. Regarding QTI, LBA had lower levels of leadership, helpful/friendly, understanding and student responsibility/freedom than NLBA.

**Table 2:** Comparison of aggression, EPQ, FES and QTI in two groups

Variable	LBA (*n* = 1078) Mean ± SD	NLBA (*n* = 3452) Mean ± SD	*P*-value
Aggression	6.33 ± 6.35	5.78 ± 6.16	0.011
EPQ			
Extraversion	16.62 ± 4.78	16.98 ± 4.66	0.026
Psychoticism	3.92 ± 3.42	3.54 ± 3.25	0.001
Neuroticism	8.72 ± 5.46	8.20 ± 5.48	0.007
Lie	14.55 ± 3.89	14.83 ± 3.75	0.034
FES			
Cohesion	6.91 ± 1.98	7.133 ± 1.93	0.001
Conflict	2.47 ± 1.94	2.28 ± 1.83	0.004
Achievement	8.17 ± 1.42	8.29 ± 1.43	0.018
Intellectual-Cultural	4.65 ± 1.86	4.73 ± 1.89	0.245
Active-Recreational	3.45 ± 2.04	3.51 ± 2.07	0.391
Organization	6.21 ± 1.99	6.31 ± 1.96	0.172
Control	4.00 ± 1.80	3.86 ± 1.81	0.021
QTI			
Leadership	17.69 ± 4.28	18.37 ± 3.95	0.000
Helpful/Friendly	16.34 ± 5.30	16.99 ± 5.11	0.000
Understanding	19.09 ± 4.42	19.73 ± 4.27	0.000
Student responsibility/Freedom	11.86 ± 4.19	12.29 ± 4.25	0.004
Uncertainty	6.20 ± 3.92	6.21 ± 4.34	0.932
Dissatisfaction	4.70 ± 4.88	4.77 ± 5.32	0.686
Admonishing	7.67 ± 4.00	7.80 ± 4.18	0.388
Strict behavior	14.51 ± 4.63	14.81 ± 4.53	0.066

*Abbreviation*: *SD* Standard Deviation, *EPQ* Eysenck Personality Questionnaire, *FES* Family Environment Scale, *QTI* Questionnaire on Teacher Interaction

As shown in Table 3, all the subscales of personality had significant correlations with aggression in both groups except for extraversion in LBA. For FES, only achievement in LBA and control in NLBA had no significant correlations with aggression (*p* > 0.05). For QTI, leadership, helpful/friendly and understanding were negatively correlated with aggression in both groups; uncertainty, dissatisfaction and admonishing were positively correlated with aggression in LBA added strict behavior in NLBA.

**Table 3:** Correlation between aggression and EPQ, FES and QTI in two groups

Variable	LBA (*n* = 1078)	NLBA (*n* = 3452)
EPQ		
Extraversion	-0.056	-0.086**
Psychoticism	0.394**	0.367**
Neuroticism	0.452**	0.410**
Lie	-0.341**	-0.332**
FES		
Cohesion	-0.260**	-0.337**
Conflict	0.260**	0.283**
Achievement	-0.043	-0.049**
Intellectual-Cultural	-0.138**	-0.147**
Active-Recreational	-0.115**	-0.122**
Organization	-0.306**	-0.281**
Control	-0.089**	-0.024
QTI		
Leadership	-0.197**	-0.187**
Helpful/Friendly	-0.167**	-0.152**
Understanding	-0.206**	-0.205**
Student Responsibility/Freedom	-0.038	0.001
Uncertainty	0.265**	0.290**
Dissatisfaction	0.311**	0.311**
Admonishing	0.237**	0.303**
Strict behavior	0.021	0.052**

Fixed factor: age, gender, SES and only child

^*^P < 0.05, **P < 0.01

Multiple linear regression models of aggressive behaviors with enter analysis for each group were made. Only significant results were shown in multiple linear regression models. As presented in Table 4, after adjusting for the confounders, aggression positively associated with, in Beta sequence, high neuroticism (Beta = 0.281, *p* < 0.001), psychoticism (Beta = 0.121, *p* < 0.001), uncertainty (Beta = 0.086, *p* = 0.004) and dissatisfaction (Beta = 0.063, *p* = 0.059) whereas negatively associated with high lie (Beta = -0.092, *p* = 0.005) and organization (Beta = -0.078, *p* = 0.012) in LBA; For NLBA, aggression positively associated with high neuroticism (Beta = 0.225, *p* < 0.001), uncertainty (Beta = 0.124, *p* < 0.001), conflict (Beta = 0.071, *p* < 0.001), dissatisfaction (Beta = 0.067, *p* = 0.045), achievement (Beta = 0.047, *p* = 0.002) and psychoticism (Beta = 0.046, *p* = 0.015), whereas negatively associated with lie (Beta = -0.129, *p* < 0.001) and cohesion (Beta = -0.126, *p* < 0.001). The value of adjusted R^2^ was 0.266 for LBA and 0.268 for NLBA.

**Table 4:** Multiple linear regression models of aggressive behaviors with enter analysis

Variables	**LBA (*n* = 1078)**			**NLBA (*n* = 3452)**		
	Beta	*P* value	R^2^	Beta	*P* value	R^2^
**Personality**						
Psychoticism	0.121	0.000		0.046	0.021	
Neuroticism	0.281	0.000		0.225	0.000	
Lie	-0.092	0.005		-0.129	0.000	
**Family environment**						
Cohesion				-0.126	0.000	
Conflict				0.071	0.000	
Achievement				0.047	0.003	
Organization	-0.078	0.012				
**Classroom environment**						
Uncertainty	0.086	0.007		0.124	0.000	
Dissatisfaction	0.063	0.068		0.067	0.000	
			0.266			0.268

Gender, age, SES and only child were fixed in the model

## Discussion

This study attempted to comprehensively examine the prevalence of aggressive behaviors in Chinese LBA and compare important psychosocial associated factors with NLBA. Our findings revealed that LBA had a slightly more serious aggressive behavioral problems than NLBA, consistent with our first hypothesis, also consistent with previous research [37, 38]. Children and adolescents need care, love, attention, and supervision of their parents even though their role can be temporally and partially replaced by grandparents or other caregivers [4]. Studies have found that children with absent parents had experienced more difficulties at school and less prosocial behaviors [39]. Abnormal behaviors were also found in LBA as part of their way of getting attention from their caregiver and to suppress their loneliness [40]. One recent systematic review of 111 studies (91 studies were done in China) including 106,167 LBA concluded that LBA had increased risk of conduct disorders comparing with NLBA [41].

As for the associated factors, our study found that individuals with high neuroticism and psychoticism tented to experience high levels of aggression, and with high lie had less aggressive behaviors, consistent with previous findings [14, 42]. Neuroticism was the strongest factor no matter in LBA or NLBA. It involves a pattern of anxiety, worrying and distress. Individuals with high neuroticism tend to have high levels of emotional instability and feel difficult in controlling impulsive and violent behaviors [19]. It has been reported that adolescents with neuroticism personality tend to be more hostile [43] and deviate from societal norms [44]. This study also found the positive effect of psychoticism on adolescents’ aggressive behaviors. The psychoticism symptom was considered as the heart of impulsivity and lack of socialization. It means traits of immaturity, tough-mindedness and anti-authoritative attitudes and then has positive effects on the daily hostility [45]. Youth with psychoticism symptoms are vulnerable to adverse mental health outcomes over time [46, 47], and even marks anti-social behaviors and later criminals [48]. It was a good predictor of implicit aggressions in Chinese adolescents and independent associations of psychoticism were also observed with children’s attention-deficit/hyperactivity disorder (ADHD) [49] and school achievement [50]. Therefore, we have reason to believe that focusing on personality characteristic trainings to control and manage aggressive behaviors in adolescents would be effective.

As for environment, our study has explored more interesting findings. First, family environment had strong effects on NLBA. Especially for cohesion, it could obviously influence aggression, consistent with previous studies [51]. Achievement, however, could slightly increase aggressive behaviors. To explain this finding, it might be said that the pursuit of success could build stressful family atmosphere and lead to aggression. One study suggested that relaxing academic standards could increase students’ happiness [21]. It was worth mentioning that, for LBA, however, the effects of family markedly reduced. LBA in our study were under a relatively poorer family environment (less cohesion and achievement, more conflict and control in our study) than NLBA. It indicated that the family natural function of LBA had broken to some degree due to the lack of parents’ companionship, and following the weakening parent-adolescent bonding and reduced parenting quality, consistent with previous findings [52]. It was considered that children in poor quality of family atmosphere were more likely to appear antisocial behaviors and easier to experience neglecting [12].

Though family function on socialization has weakened in LBA, the effect of classroom environment still existed. This finding was in line with our hypothesis that different types of environments had different effects on aggression between LBA and NLBA. The teacher style of dissatisfaction could make for aggressive behaviors both in LBA and NLBA. Communications and supports from significant adults can prevent adolescents from experiencing behavioral problems [7]. Likewise, a poor classroom environment was an important predictor of adolescents’ externalizing behavior problems [53]. Class head teachers play an indispensable adult role in adolescents’ lives, especially in LBA. Teachers are key figures in students’ behavioral development since one research revealed that students’ willingness to ask their head teacher for help was associated with a 49% decrease of suicidal tendency and school and class interpersonal relationships were key determinants of students’ mental health [54]. Unfortunately, LBA in our study had a relatively disadvantaged classroom environment and poor teacher-student interaction. But at the same time, focusing on class environment maybe more practical than on family environment for LBA to cope with their problems due to the lack of parenting for them. Schools were suitable settings for taking promotion interventions in left-behind situation comparing with more unpredictable family supports. Previous studies have also given the same suggestion [55]. In this way, improving classroom environment in LBA might be a feasible and operable approach to effectively decrease aggressive behaviors in LBA.

This study has some limitations. First, the subjects of this study were adolescents from Liaoning and Anhui provinces, which can't represent the entire adolescent population in China. Although the population size and average income of the two provinces can respectively represent the average level of northern and southern China, and the larger sample size is also somewhat representative. Second, our cross-sectional design prevented us from examining causal relationships among these factors and further longitudinal studies are needed to examine how these factors of aggressive behaviors works. Third, some details for parent absence, such as age at separation and the frequency of communication may potentially affect LBA’s behavior, and need to be explored in future studies. Fourth, despite the significant influence of classroom environment on adolescents’ aggressive behaviors, other class factors may also represent the classroom climate, such as peer relationship and class routines. Moreover, some school factors also need to be considered in the future study since the feasible intervention setting for school and class. Further research should consider it and perform a more precise control study. Finally, it’s hard to avoid the bias of responses in the process of children's investigation, so we try our best to reduce the bias in the process of questionnaire design and investigation and analysis, such as selecting questionnaires suitable for 10–18 years old, answering doubts in time during the investigation, and doing reliability analysis during the analysis to ensure reliability. Despite these limitations, the researchers believe that these findings, which are based on a fairly large and representative sample, offer some important information regarding aggressive behaviors among Chinese LBA.

## Conclusion

These findings revealed new insights into the nature of LBA’ aggressive behaviors in Chinese left-behind context and highlighted the fact that LBA had different effects of associated factors on aggressive behavior from that in NLBA. Cultivating favorable personality was the most important way for adolescents. Promoting cohesion and lessen conflict between family members were still effective for NLBA. Schools and families should pay attention to the personality characteristics of LBA, and shape pleasant and cheerful and popular personality traits by opening corresponding psychological courses and carrying out corresponding psychological counseling in a planned manner, reducing the personality level of psychoticism, neuroticism and lie, and improving their emotional management ability. More importantly, since the separation of parents may temporarily weaken their family functions, it is more operable and effective to create a good classroom environment, cultivate a harmonious relationship between teachers, students, and classmates, enhance the attention and guidance to the emotional and behavioral problems of LBA, strengthen their protection and support functions, and cultivate their correct behaviors.

## Data Availability

The data are not publicly available because they contain information that could compromise research participant privacy and consent, but are available from the corresponding author upon reasonable request.
